# Imipenem-Relebactam Susceptibility in *Enterobacterales* Isolates Recovered from ICU Patients from Spain and Portugal (SUPERIOR and STEP Studies)

**DOI:** 10.1128/spectrum.02927-22

**Published:** 2022-08-31

**Authors:** Marta Hernández-García, María García-Castillo, Germán Bou, Emilia Cercenado, Mercedes Delgado-Valverde, Antonio Oliver, Cristina Pitart, Jesús Rodríguez-Lozano, Nuria Tormo, José Melo-Cristino, Margarida F. Pinto, Elsa Gonçalves, Valquíria Alves, Ana Raquel Vieira, Elmano Ramalheira, Luísa Sancho, José Diogo, Rui Ferreira, Hugo Cruz, Catarina Chaves, Joana Duarte, Leonor Pássaro, Jazmín Díaz-Regañón, Rafael Cantón

**Affiliations:** a Servicio de Microbiología, Hospital Universitario Ramón y Cajal –IRYCIS, Madrid, Spain; b CIBER de Enfermedades Infecciosas (CIBERINFEC), Instituto de Salud Carlos III, Madrid, Spain; c Servicio de Microbiología, Hospital Universitario A Coruña, A Coruña, Spain; d Servicio de Microbiología Clínica y Enfermedades Infecciosas, Hospital General Universitario Gregorio Marañón, Madrid, Spain; e Unidad Clínica de Enfermedades Infecciosas y Microbiología, Instituto de Biomedicina de Sevilla (IBIS), Hospital Universitario Virgen Macarena/CSIC/Universidad de Sevilla, Sevilla, Spain; f Servicio de Microbiología, Hospital Universitario Son Espases, Palma de Mallorca, Spain; g Servicio de Microbiología, Hospital Clínic i Provincial, Barcelona, Spain; h Servicio de Microbiología, Hospital Universitario Marqués de Valdecilla, Santander, Spain; i Servicio de Microbiología, Consorcio Hospital General Universitario de Valencia, Valencia, Spain; j Laboratório de Microbiologia, Centro Hospitalar Universitário Lisboa Norte, Lisboa, Portugal; k Laboratório de Microbiologia, Serviço de Patologia Clínica, Centro Hospitalar Universitário Lisboa Central, Lisboa, Portugal; l Laboratório de Microbiologia Clínica, Centro Hospitalar de Lisboa Ocidental, Lisboa, Portugal; m Laboratório de Microbiologia, Unidade Local de Saúde de Matosinhos, Matosinhos, Portugal; n Serviço de Patologia Clínica, Centro Hospitalar Universitário São João, Porto, Portugal; o Serviço Patologia Clínica, Hospital Infante Dom Pedro, Aveiro, Portugal; p Serviço de Patologia Clínica, Hospital Professor Fernando da Fonseca, Amadora, Portugal; q Serviço de Microbiologia, Hospital Garcia de Orta, Almada, Portugal; r Serviço de Patologia Clínica–Microbiologia, CHUA–Unidade de Portimão, Portimão, Portugal; s Serviço de Microbiologia, Centro Hospitalar Universitário do Porto, Porto, Portugal; t Serviço de Microbiologia, Centro Hospitalar Universitário de Coimbra, Coimbra, Portugal; u MSD Portugal, Paço de Arcos, Portugal; v Departamento Médico, MSD España, Madrid, Spain; Universidad de Buenos Aires, Facultad de Farmacia y Bioquímica

**Keywords:** imipenem-relebactam susceptibility, carbapenemase-producing *Enterobacterales*, ICU patients, Spain, Portugal

## Abstract

Imipenem-relebactam is a novel β-lactam-β-lactamase inhibitor combination. We evaluated the *in vitro* activity of imipenem-relebactam and comparators against *Enterobacterales* clinical isolates recovered in 8 Spanish and 11 Portuguese intensive care units (ICUs) (SUPERIOR, 2016–2017; STEP, 2017–2018). Overall, 747 *Enterobacterales* isolates (378 Escherichia coli, 252 Klebsiella spp., 64 Enterobacter spp., and 53 other species) were prospectively collected from ICU patients with complicated intraabdominal (cIAI), complicated urinary tract (cUTI), and lower respiratory tract (LRTI) infections. MICs were determined (ISO-broth microdilution), and whole-genome sequencing (WGS) was performed in a subset of isolates displaying susceptible and resistant imipenem-relebactam MICs. Imipenem-relebactam (98.7% susceptible) showed similar activity to ceftazidime-avibactam (99.5% susceptible) and higher than ceftolozane-tazobactam (86.9% susceptible). Imipenem-relebactam was inactive against 1.3% (10/747) isolates, all of them due to carbapenemase production (9 K. pneumoniae and 1 E. cloacae). Imipenem-relebactam was active against 100% of extended-spectrum β-lactamase (ESBL)-E. coli and ESBL-Klebsiella spp. isolates and 80.4% of carbapenemase-Klebsiella spp. producers. Carbapenemase genes were confirmed by WGS in 41 Klebsiella spp.: OXA-48 (20/41), KPC-3 (14/41), OXA-181 (4/41), NDM-1 (1/41), OXA-48 + VIM-2 (1/41), and KPC-3 + VIM-2 (1/41). In Klebsiella spp. isolates, relebactam restored imipenem susceptibility in all KPC-3 producers, and resistant isolates (7/41) were mostly OXA-48 + CTX-M-15-K. pneumoniae high-risk clones (7/9). Intercountry differences were detected as follows: OXA-48 (17/21) was dominant in Spain, unlike KPC-3 (14/15) in Portugal. Imipenem-relebactam was 100% active against CTX-M-15-ST131-H30Rx-E. coli high-risk clone, predominant in both countries. Our results depict the potential role of imipenem-relebactam in ICU patients with cIAIs, cUTIs, and LRTIs due to wild-type ESBL- and carbapenemase-producing *Enterobacterales*, particularly KPC producers.

**IMPORTANCE** We comparatively evaluate the *in vitro* activity of a drug combination consisting of a carbapenem (imipenem) and a novel inhibitor of beta-lactamases (relebactam), a mechanism that destroys beta-lactam antibiotics. We assess the activity against a collection of *Enterobacterales* clinical isolates recovered from difficult-to-treat infections in patients admitted to different intensive care units in Portugal and Spain. Imipenem-relebactam shows excellent activity in avoiding common resistance mechanisms in this setting, such as extended-spectrum beta-lactamases and carbapenemases widely distributed, including KPCs. We show few resistant isolates (<2%). Molecular characterization by whole-genome sequencing shows that most of the resistant isolates produced specific carbapenemase, such as OXA-48 or metalo-betalactamases. Our study updates the activity of imipenem-relebactam in light of current epidemiology in a hospital setting in which the use of this combination is needed due to the presence of infections due to multidrug-resistant isolates.

## INTRODUCTION

The increasing rate of infections due to carbapenem-resistant *Enterobacterales* is a major public health concern and has led to serious therapeutic challenges in the clinical setting. In response, the development of novel β-lactam-β-lactamase inhibitor combinations active against these multidrug-resistant pathogens has become a priority (1, 2). Relebactam (formerly known as MK-7655) is a new β-lactamase inhibitor that combined with imipenem-cilastatin shows promising activity against certain carbapenem-resistant *Enterobacterales* strains (3, 4). Production of carbapenemases is the most frequent resistance mechanism to carbapenems; most clinically significant of these enzymes belong to class A (KPC), class B (metallo-β-lactamases or [MBL]), and class D (OXA-48-*like*) (5, 6). In *Enterobacterales*, imipenem-relebactam has potent activity against a wide range of β-lactamases, including class A (such as KPC carbapenemases and extended-spectrum β-lactamases [ESBLs]), class C (AmpC), and certain D β-lactamases other than OXA-48 enzyme (3, 4, 7).

Between 2019 and 2020, the clinical use of imipenem-relebactam was approved for the treatment of complicated urinary tract infections (cUTIs) and complicated intra-abdominal infections (cIAIs) by the FDA and for hospital-acquired and ventilator-associated bacterial pneumonia by the EMA in adult patients with limited or no alternative therapeutic options (4, 8). In intensive care units (ICUs), the high prescription of broad-spectrum antibiotics to treat patients with severe infections leads to high rates of antimicrobial resistance, resulting in increased mortality and morbidity rates, prolonged hospital stays, and high costs to the health care system (9, 10). The appropriate use of available antibiotics and the correct implementation of novel β-lactam-β-lactamase inhibitor combinations, such as imipenem-relebactam, in the treatment of severe infections can help reduce and prevent high rates of multidrug resistance due to CRE in hospital units with critically ill patients, such as ICUs.

This study aimed to evaluate the *in vitro* activity of imipenem-relebactam and comparator agents (ceftazidime-avibactam and ceftolozane-tazobactam) against relevant clinical *Enterobacterales* isolates collected prospectively in Spain and Portugal from ICU patients with cIAI, cUTI, and lower respiratory tract infections (LRTI) as a part of two surveillance studies (SUPERIOR and STEP). Additionally, we studied the whole-genome sequencing (WGS), the molecular epidemiology, and the resistome of a subset of isolates, focusing on the resistance mechanisms compromising imipenem-relebactam efficacy.

## RESULTS

### Bacterial isolates.

A total of 747 *Enterobacterales* (Spain [*n* = 359] and Portugal [*n* = 388]) nonreplicate clinical isolates were recovered from cUTI (Spain [57.9%, 208/359], Portugal [49.2%, 191/388]), cIAI (Spain [42.1%, 151/359], Portugal [28.6%, 111/388]), and LRTI (Portugal [22.2%, 86/388]). E. coli was the most frequent species in both countries, followed by Klebsiella pneumoniae and Enterobacter cloacae. Other minority *Enterobacterales* species were also found in both countries (Table S1 in the supplemental material).

### Antimicrobial susceptibility testing results.

In this collection, imipenem-relebactam (98.7% S by EUCAST and 96.4% S by CLSI; MIC_50_ = 0.12/4 mg/liter; MIC_90_ = 0.5/4 mg/liter) and ceftazidime-avibactam (99.5% S by EUCAST and CLSI; MIC_50_ = 0.12/4 mg/liter; MIC_90_ = 0.5/4 mg/liter) showed a similar activity, followed by ceftolozane-tazobactam (86.9% S by EUCAST and CLSI; MIC_50_ = 0.25/4 mg/liter; MIC_90_ = 8/4 mg/liter) (Table 1). Differences in susceptibility rates of imipenem-relebactam (Spain [97.8% S by EUCAST and 93.9% S by CLSI]; Portugal [99.5% S by EUCAST and 98.7% S by CLSI]) and comparators by country were not found (Table 2).

**TABLE 1:** Antimicrobial activity of imipenem-relebactam and comparators against *Enterobacterales* during the SUPERIOR and STEP surveillance studies broken down by major organisms and phenotypes

Organisms (no. tested)/antimicrobials				EUCAST						CLSI					
				S^*b*^		I		R		S		I		R	
	MIC_50_	MIC_90_	Range	No.	%	No.	%	No.	%	No.	%	No.	%	No.	%
All *Enterobacterales* (*n* = 747)															
CZA^*a*^	0.12	0.5	≤0.03 to >64	743	99.5			4	0.5	743	99.5			4	0.5
CTZ^*a*^	0.25	8	0.12 to >32	649	86.9			98	13.1	649	86.9	14	1.9	84	11.2
IMR^*a*^	0.12	0.5	0.06 to >64	737	98.7			10	1.3	720	96.4	17	2.3	10	1.3
IMI	0.12	1	0.06 to -64	718	96.1	7	0.9	22	2.9	692	92.6	26	3.5	29	3.9
All E. coli (*n* = 378)															
CZA	0.12	0.25	≤0.03 to >64	377	99.7			1	0.3	377	99.7			1	0.3
CTZ	0.25	1	0.12 to >32	368	97.4			10	2.6	368	97.4	2	0.5	8	2.1
IMR	0.12	0.25	0.06 to 2	378	100			0	0	377	99.7	1	0.3	0	0
IMI	0.12	0.25	0.06 to 8	376	99.5	1	0.3	1	0.3	375	99.2	1	0.3	2	0.5
ESBL^*c*^ E. coli (*n* = 75)															
CZA	0.25	0.5	≤0.03 to >64	74	98.7			1	1.3	74	98.7			1	1.3
CTZ	0.5	4	0.12 to >32	67	89.3			8	10.7	67	89.3	1	1.3	7	9.3
IMR	0.12	0.25	0.12 to 1	75	100			0	0	75	100	0	0	0	0
IMI	0.12	0.25	0.12 to 1	75	100	0	0	0	0	75	100	0	0	0	0
All Klebsiella spp.^*d*^ (*n* = 252)															
CZA	0.25	1	≤0.03 to >64	251	99.6			1	0.4	251	99.6			1	0.4
CTZ	0.5	>32	0.12 to >32	183	72.6			69	27.4	183	72.6	10	4.0	59	23.4
IMR	0.25	1	0.06 to >64	243	96.4			9	3.6	235	93.3	8	3.2	9	3.6
IMI	0.12	2	0.06 to 64	227	90.1	5	2.0	20	7.9	212	84.1	15	6.0	25	9.9
ESBL^*c*^ Klebsiella spp. (*n* = 66)															
CZA	0.25	1	0.12 to 1	66	100			0	0	66	100			0	0
CTZ	1	16	0.25 to >32	45	68.2			21	31.8	45	68.2	5	7.6	16	24.2
IMR	0.12	0.5	0.06 to 0.25	66	100			0	0	66	100	0	0	0	0
IMI	0.12	0.5	0.06 to 1	66	100	0	0	0	0	66	100	0	0	0	0
CP^*e*^ Klebsiella spp. (*n* = 46)															
CZA	0.5	2	0.12 to >64	45	97.8			1	2.2	45	97.8			1	2.2
CTZ	>32	>32	0.25 to >32	7	15.2			39	84.8	7	15.2	2	4.3	37	80.4
IMR	0.5	64	0.06 to >64	37	80.4			9	19.6	29	63.0	8	17.4	9	19.6
IMI	4	32	0.12 to 64	22	47.8	5	10.9	19	41.3	10	21.7	12	26.1	24	52.2
All *Enterobater* spp.^*f*^ (*n* = 64)															
CZA	0.25	1	≤0.03 to >64	62	96.9			2	3.1	62	96.9			2	3.1
CTZ	0.5	>32	0.12 to >32	48	75.0			16	25.0	48	75.0	2	3.1	14	21.9
IMR	0.25	0.25	0.06 to 16	63	98.4			1	1.6	63	98.4	0	0	1	1.6
IMI	0.5	1	0.12 to 16	62	96.9	1	1.6	1	1.6	60	93.8	2	3.1	2	3.1
All *Serratia* spp.^*g*^ (*n* = 24)															
CZA	0.25	0.5	0.12 to 1	24	100			0	0	24	100			0	0
CTZ	0.5	2	0.5 to 2	24	100			0	0	24	100	0	0	0	0
IMR	1	2	0.25 to 2	24	100			0	0	17	70.8	7	29.2	0	0
IMI	1	2	0.25 to 2	24	100	0	0	0	0	17	70.8	7	29.2	0	0
All *Citrobacter* spp.^*h*^ (*n* = 19)															
CZA	0.12	0.25	0.06 to 0.5	19	100			0	0	19	100			0	0
CTZ	0.25	8	0.12 to 16	16	84.2			3	15.8	16	84.2	0	0	3	15.8
IMR	0.12	0.25	0.06 to 0.25	19	100			0	0	19	100	0	0	0	0
IMI	0.25	1	0.06 to 1	19	100	0	0	0	0	19	100	0	0	0	0
All other *Enterobacterales* spp.^*i*^ (*n* = 10)															
CZA	0.25	0.5	0.12 to 2	10	100			0	0	10	100			0	0
CTZ	0.5	2	0.25 to 2	10	100			0	0	10	100	0	0	0	0
IMR	0.25	1	0.12 to 2	10	100			0	0	9	90	1	10	0	0
IMI	0.5	1	0.12 to 2	10	100	0	0	0	0	9	90	1	10	0	0

a Imipenem-relebactam (IMR), ceftazidime-avibactam (CZA) and ceftolozane-tazobactam (CTZ) were tested with a fixed concentration of 4 mg/liter of relebactam, avibactam and tazobactam.

b S/I/R, susceptible, standard dose/susceptible, increased exposure/resistant, by EUCAST; susceptible/intermediate/resistant, by CLSI.

c ESBL phenotype (MICs ≥ 2 mg/liter for cefotaxime, ceftazidime and/or cefepime).

d Klebsiella spp. group includes K. pneumoniae (*n* = 211), K. aerogenes (*n* = 25), K. oxytoca (*n* = 13), K. variicola (*n* = 2), and Raoultella ornithinolytica (*n* = 1).

e Carbapenemase (CP) phenotype (MICs > 1 mg/liter for imipenem and/or > 0.12 mg/liter for meropenem).

f Enterobacter spp. group includes E. cloacae (*n* = 60), *E. asburiae* (*n* = 2), *E. kobei* (*n* = 1), and *E. hormaechei* (*n* = 1).

g *Serratia* spp. group includes S. marcescens (*n* = 23) and S. liquefasciens (*n* = 1).

h *Citrobacter* spp. group includes C. koseri (*n* = 10), C. braakii (*n* = 4), C. freundii (*n* = 4), and C. sakazakii (*n* = 1).

i Other *Enterobacterales* spp. group includes Hafnia alvei (*n* = 5), Providencia stuartii (*n* = 5), Kluyvera ascorbata (*n* = 1), and Salmonella enterica (*n* = 1).

**TABLE 2:** Antimicrobial activity of imipenem-relebactam and comparators against *Enterobacterales* during the SUPERIOR and STEP surveillance studies broken down by countries

All enterobacterales (no. tested)/antimicrobials				EUCAST						CLSI					
				S^*b*^		I		R		S		I		R	
	MIC_50_	MIC_90_	Range	No.	%	No.	%	No.	%	No.	%	No.	%	No.	%
All *Enterobacterales* in Spain (*n* = 359, SUPERIOR)															
CZA^*a*^	0.12	0.5	≤0.03 to >64	356	99.2			3	0.8	356	99.2			3	0.8
CTZ^*a*^	0.25	8	0.12 to >32	314	87.5			45	12.5	314	87.5	5	1.4	40	11.1
IMR^*a*^	0.12	0.5	0.06 to >64	351	97.8			8	2.2	337	93.9	14	3.9	8	2.2
IMI	0.12	1	0.06 to 64	351	97.8	2	0.6	6	1.7	334	93.0	17	4.7	8	2.2
All *Enterobacterales* in Portugal (*n* = 388, STEP)															
CZA	0.12	0.5	≤0.03 to >64	387	99.7			1	0.3	387	99.7			1	0.3
CTZ	0.25	8	0.12 to >32	335	86.3			53	13.7	335	86.3	9	2.3	44	11.3
IMR	0.12	0.5	0.06 to 64	386	99.5			2	0.5	383	98.7	3	0.8	2	0.5
IMI	0.12	1	0.06 to 64	367	94.6	5	1.3	16	4.1	358	92.3	9	2.3	21	5.4

a Imipenem-relebactam (IMR), ceftazidime-avibactam (CZA), and ceftolozane-tazobactam (CTZ) were tested with a fixed concentration of 4 mg/liter of relebactam, avibactam and tazobactam.

b S/I/R, susceptible, standard dose/susceptible, increased exposure/resistant, by EUCAST; susceptible/intermediate/resistant, by CLSI.

**FIG 1:**
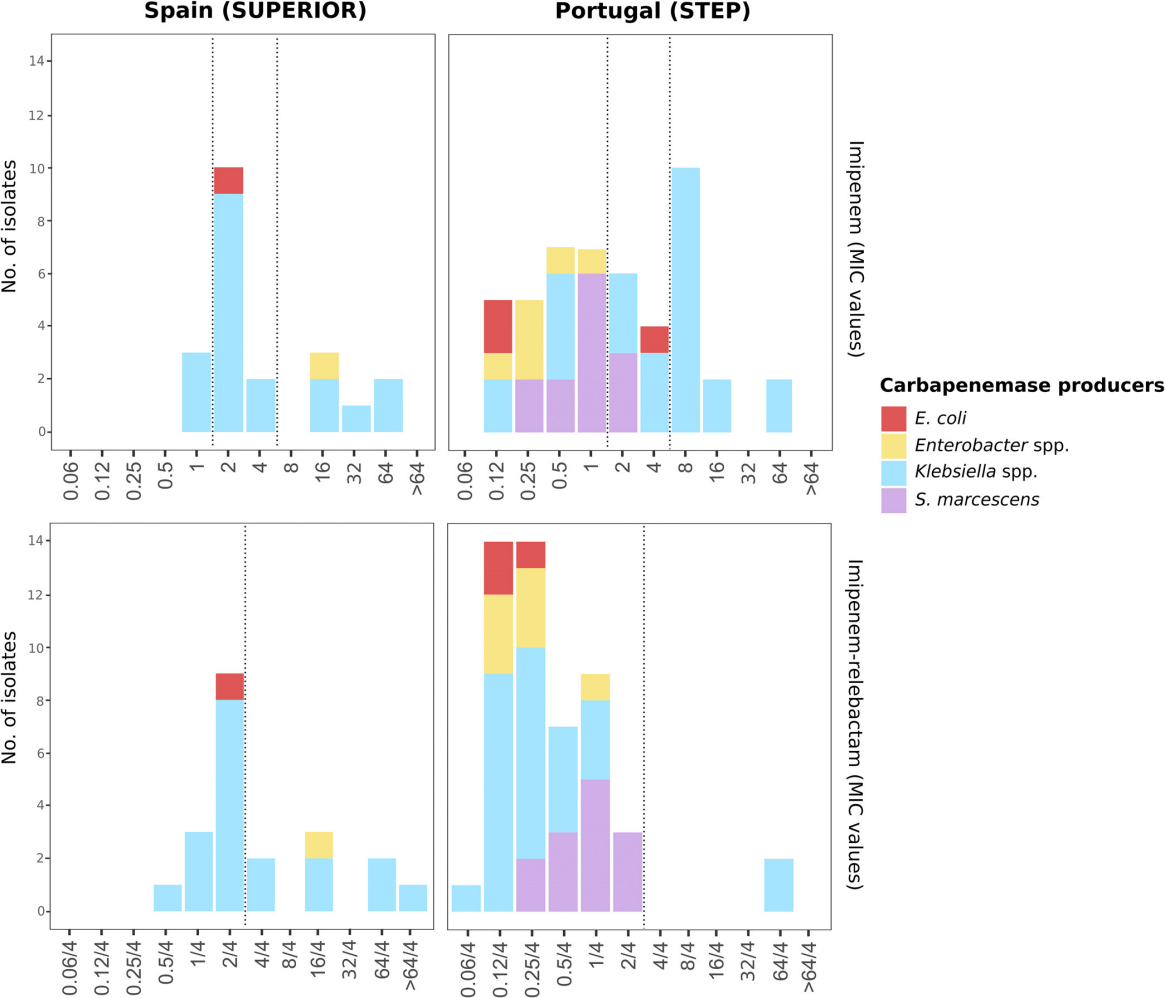
Distribution of *Enterobacterales* isolates with a carbapenemase phenotype (*n* = 69) recovered during the SUPERIOR and STEP surveillance studies by country and the MIC value of imipenem-relebactam and imipenem. Dotted lines represent the EUCAST clinical breakpoints of imipenem-relebactam (S, MIC ≤ 2/4 mg/liter; R, MIC > 2/4 mg/liter) and imipenem (S, MIC ≤ 1 mg/liter; R, MIC > 4 mg/liter).

Overall, the imipenem-relebactam resistance rate was 1.3% (10/747) (2.2% in Spain; 0.5% in Portugal) by both EUCAST and CLSI criteria (Table 1 and 2). All imipenem-relebactam-resistant strains (MIC_IMR_ = 4 to >64 mg/liter; 9 K. pneumoniae, and 1 E. cloacae) were phenotypically classified as carbapenemase producers. The distribution of all *Enterobacterales* isolates with a carbapenemase phenotype (*n* = 69) by countries, species, and MIC values of imipenem-relebactam and imipenem is shown in Fig. 1.

Based on EUCAST breakpoints, the susceptibility of imipenem-relebactam against E. coli isolates (*n* = 378) (100% S) was comparable to that of ceftazidime-avibactam (99.7% S) and ceftolozane-tazobactam (97.4% S). Against the subset of ESBL-E. coli isolates (19.8%, 75/378), the activity of imipenem-relebactam (100% S) was slightly higher than ceftazidime-avibactam (98.7% S) and ceftolozane-tazobactam (89.3% S) (Table 1).

Among the collection of Klebsiella spp. isolates (*n* = 252), imipenem-relebactam (96.4% S) and ceftazidime-avibactam (99.6% S) displayed similar activity, followed by ceftolozane-tazobactam (72.6% S). In the subset of Klebsiella spp. isolates with a phenotype compatible with ESBL production (26.2%, 66/252), the susceptibility rate of imipenem-relebactam (100% S) was comparable to ceftazidime-avibactam (100% S) and higher than ceftolozane-tazobactam (68.2% S). Against the Klebsiella spp. isolates with a carbapenemase phenotype (18.2%, 46/252), the most active antimicrobial combination was ceftazidime-avibactam (97.8% S), followed by imipenem-relebactam (80.4% S) and ceftolozane-tazobactam (15.2% S). In addition, among carbapenemase-producing Klebsiella strains, imipenem activity did not exceed 60% (47.8% categorized as susceptible, standard dose [S] and 10.9% as susceptible, increased exposure [I]) (Table 1).

Among the remaining *Enterobacterales* species, imipenem-relebactam was 100% active against *Serratia* spp., *Citrobacter* spp., and all isolates belonging to other minority *Enterobacterales* species and was 98.4% in Enterobacter spp. (Table 1).

Overall, relebactam reduced the imipenem MIC values at least 3-fold in 28 (3.4%) isolates, of which 16 showed a carbapenemase phenotype (14 K. pneumoniae, 1 *K. variicola*, and 1 E. coli), 4 an ESBL phenotype (2 E. cloacae, 1 *Citrobacter brakii* and 1 Citrobacter freundii), and other 8 isolates a non-ESBL/noncarbapenemase phenotype (3 E. cloacae, 2 K. pneumoniae, 2 C. freundii, and 1 E. coli). Moreover, 14 of these isolates (13 K. pneumoniae and 1 E. coli) displayed resistance to imipenem but susceptibility to imipenem-relebactam according to the EUCAST criteria (MIC_IMP_ = 8 to 16 mg/liter; MIC_IMR_ = 0.06 to 1 mg/liter).

In addition, activity of imipenem-relebactam (MIC_IMR_ ≤ 2/4 mg/liter, EUCAST criteria) was excellent regardless the source of infection: cUTI (99.0% S), cIAI (98.1% S), and LRTI (98.8% S) and was especially potent against Klebsiella spp. isolates with carbapenemase phenotype recovered from LRTI (91.7% S) (Table 3 and Fig. S1).

**TABLE 3:** Antimicrobial activity of imipenem-relebactam (number of isolates and % of susceptibility, EUCAST-2021 breakpoint) in *Enterobacterales* isolates during the SUPERIOR and STEP surveillance studies broken down by species, major phenotypes, and source of infection^*a*^

	cUTI (*n* = 399)		cIAI (*n* = 262)		LRTI (*n* = 86)		TOTAL (*n* = 747)	
Imipenem-relebactam activity	MIC ≤ 2/4 mg/liter		MIC ≤ 2/4 mg/liter		MIC ≤ 2/4 mg/liter		MIC ≤ 2/4 mg/liter	
	No.	%	No.	%	No.	%	No.	%
All *Enterobacterales*	395	99.0	257	98.1	85	98.8	737	98.7
E. coli	233	100	129	100	16	100	378	100
ESBL-E. coli	44	100	28	100	3	100	75	100
Klebsiella spp.	119	97.5	78	94.0	46	97.9	243	96.4
ESBL-Klebsiella spp.	33	100	23	100	10	100	66	100
CP-Klebsiella spp.	12	80	14	73.7	11	91.7	37	80.4
Enterobacter spp.	23	95.8	28	100	12	100	63	98.4
*Serratia* spp.	9	100	8	100	7	100	24	100
*Citrobacter* spp.	6	100	9	100	4	100	19	100
Other *Enterobacterales* spp.	5	100	5	100			10	100

a IMR, imipenem-relebactam; MIC_IMR_ ≤ 2/4 mg/liter, susceptible, standard dose by EUCAST-2021 criteria; cUTI, complicated urinary tract infection; cIAI, complicated intra-abdominal tract infection; LRTI, lower respiratory tract infection.

### Molecular epidemiology and antibiotic resistance genes.

**FIG 2:**
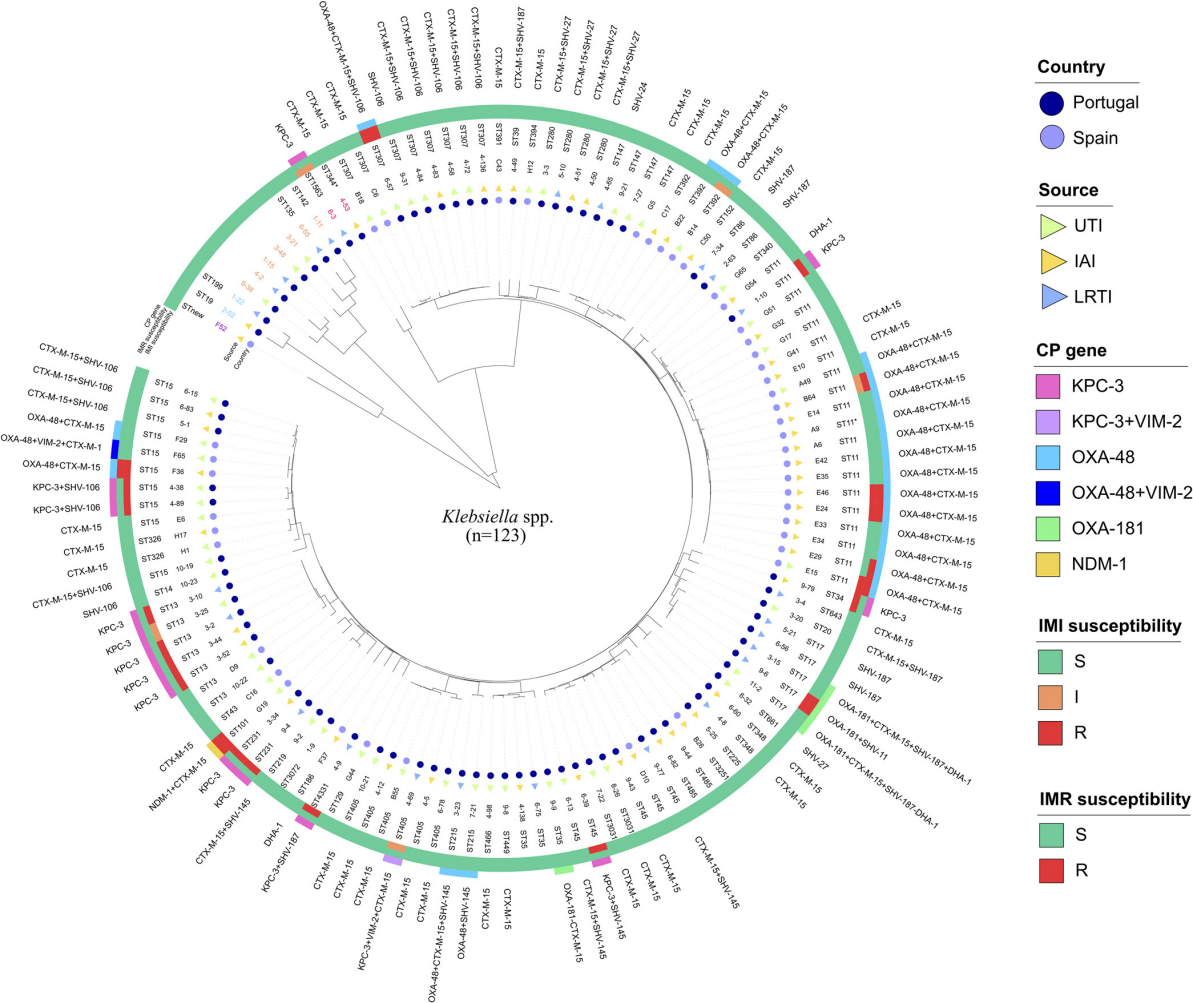
Similarity tree of Klebsiella spp. isolates sequenced (*n* = 123) during the SUPERIOR and STEP surveillance studies and molecular data (sequence type and *bla* genes content) obtained by WGS. Imipenem-relebactam (S, MIC ≤ 2/4 mg/liter; R, MIC > 2/4 mg/liter) and imipenem (S, MIC ≤ 1 mg/liter; R, MIC > 4 mg/liter) susceptibility results interpreted according to EUCAST-2021 criteria are also included. Branch length is indicative of the MASH distance. Species other than K. pneumoniae (black) are represented by colored letters: purple (*K. michiganenesis*), blue (K. oxytoca), orange (K. aerogenes), and dark pink (*K. variicola*).

Among the sequenced Klebsiella spp. isolates (*n* = 123), K. pneumoniae (111/123) was the predominant species in both countries (Spain [43/44] and Portugal [68/79]). Other species were also found in Spain (1/44 *K. michiganensis*) and Portugal (7/79 K. aerogenes, 2/79 K. oxytoca, and 2/79 *K. variicola*). Carbapenemase production was demonstrated in 41 isolates (40 K. pneumoniae and 1 *K. variicola*), although in different proportions in Spanish (43.2%, 19/44) and Portuguese (27.8%, 22/79) hospitals. OXA-48 was the most frequent carbapenemase enzyme (48.8%, 20/41) followed by KPC-3 (34.1%, 14/41), OXA-181 (7.3%, 4/41), and NDM-1 (2.4%, 1/41). In addition, the presence of multiple carbapenemase genes was also detected (KPC-3 + VIM-2 [1/41] and OXA-48 + VIM-2 [1/41]). Differences in the distribution of enzymes were also found limited to the origin country: Spain (17 OXA-48, 1 OXA-48 + VIM-2, and 1 NDM-1) and Portugal (14 KPC-3, 4 OXA-181, 3 OXA-48, and 1 KPC-3 + VIM-2) (Fig. 2 and Fig. S2). Overall, a high content of ESBL (CTX-M-15 [45/123], CTX-M-15 + SHV-*like* [22], and SHV-*like* [14/123]) and other β-lactamase genes (OXA-1 [77/123]) was also found. Clinical, epidemiological, molecular, and antimicrobial susceptibility data of all sequenced Klebsiella spp. strains are summarized in Table S2.

**FIG 3:**
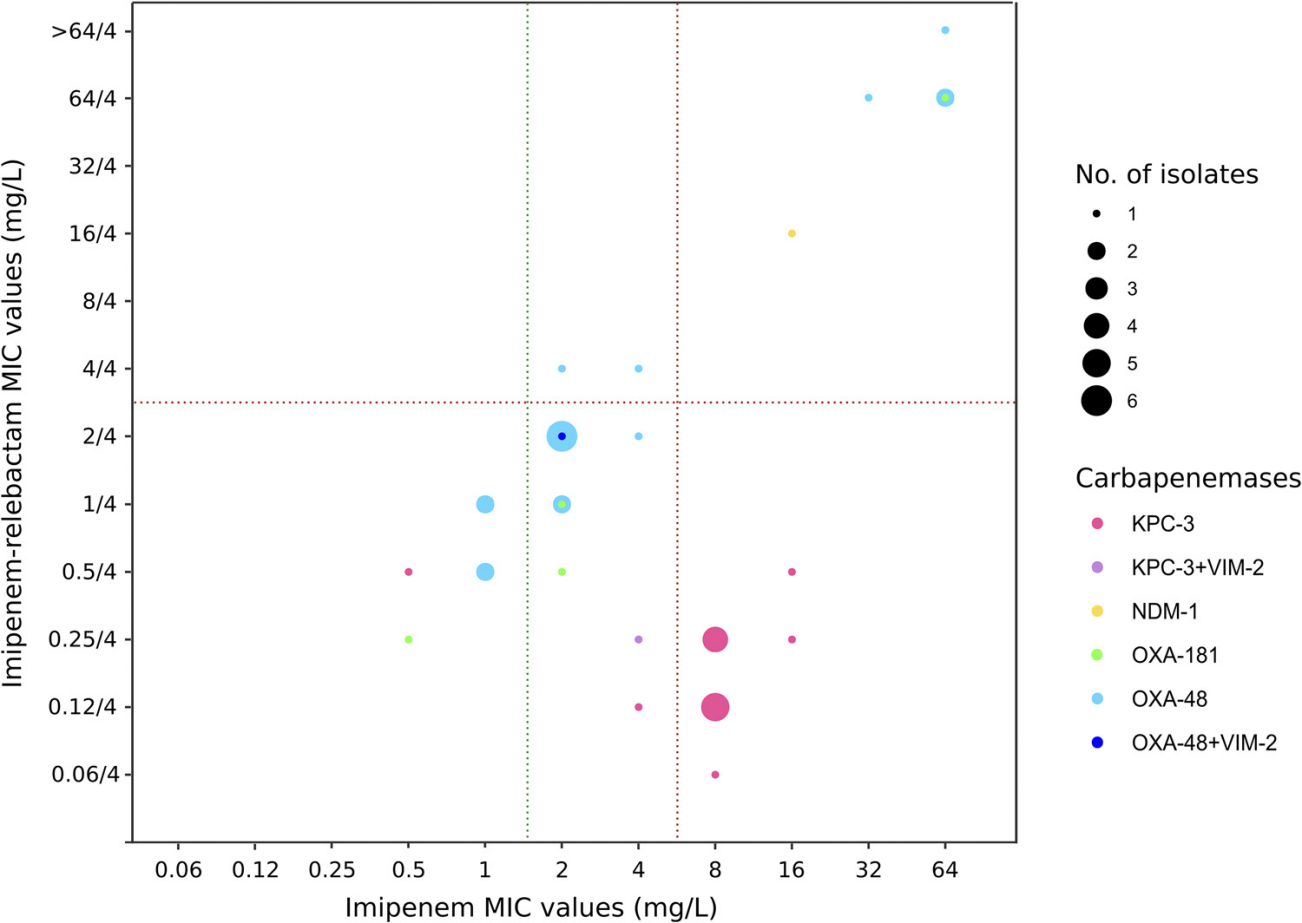
Distribution of sequenced carbapenemase-producing Klebsiella spp. isolates (*n* = 41) recovered during the SUPERIOR and STEP surveillance studies by the MIC value of imipenem-relebactam and imipenem. Dotted lines represent the EUCAST clinical breakpoints of imipenem-relebactam (S, MIC ≤ 2/4 mg/liter; R, MIC > 2/4 mg/liter) and imipenem (S, MIC 1 mg/liter; R, MIC > 4 mg/liter).

Imipenem-relebactam resistance rate (MIC_IMR_ > 2/4 mg/liter) was 7.3% (9/123) among all Klebsiella isolates and 21.9% (9/41) in the subset of carbapenemase-producing strains (Table 4). Klebsiella spp. resistant isolates belonged to high-risk clones, most of them OXA-48 producers [OXA-48 + CTX-M-15-ST11 (*n* = 5), OXA-48 + CTX-M-15-ST15 (*n* = 1), and OXA-48 + CTX-M-15-ST307 (*n* = 1)] but also NDM-1 (NDM-1 + CTX-M-15-ST101 [*n* = 1])- and OXA-181-producing strains (OXA-181 + SHV-11-ST17 [*n* = 1]). Three OXA-181 producers (75%, 3/4) (ST17 [*n* = 2] and ST35 [*n* = 1]) and up to 66.7% (14/21) of OXA-48-K. pneumoniae producers (including the OXA-48 + VIM-2 coproducer) (ST11 [*n* = 8], ST215 [*n* = 2], ST392 [*n* = 2], and ST15 [*n* = 2]) were susceptible to imipenem-relebactam (Fig. 2). To note that relebactam did not restore the activity of imipenem in these OXA-48 producers and the observed activity of imipenem-relebactam combination in these isolates is most likely related to susceptibility to imipenem itself (Fig. 3 and Table 4). However, relebactam improved the activity of imipenem by 4 to 7 dilutions in all KPC-3-producing Klebsiella spp. isolates, including the KPC-3 + VIM-2 producer (36.6%, 15/41) (12/15 categorized as imipenem resistant [R] and 3/15 as susceptible, increased exposure [I]) (Fig. 3 and Table 4).

**TABLE 4:** Antimicrobial activity of imipenem-relebactam, imipenem and ceftazidime-avibactam (EUCAST criteria) against the carbapenemase-producing Klebsiella spp. isolates recovered during the SUPERIOR and STEP surveillance studies broken down by the carbapenemase type produced

		Imipenem				Imipenem-relebactam				Ceftazidime-avibactam			
β-lactamases	ST (no. of isolates)	S + I		R		S		R		S		R	
		No.	%	No.	%	No.	%	No.	%	No.	%	No.	%
All carbapenemases (*n* = 41)		22	53.7	19	46.3	32	78.1	9	21.9	40	97.6	1	2.4
OXA-48^*a*^ (*n* = 21)	ST11 (13), ST15 (3), ST215 (2), ST392 (2), ST307 (1)	16	76.2	5	23.8	14	66.7	7	33.3	21	100	0	0
KPC-3^*b*^ (*n* = 15)	ST13 (5), ST15 (2), ST231 (2), ST11 (1), ST405 (1), ST4331 (1), ST34 (1), ST45 (1)	3	20.0	12	80.0	15	100	0	0	15	100	0	0
OXA-181 (*n* = 4)	ST17 (3), ST35 (1)	3	75	1	25	3	75	1	25	4	100	0	0
NDM-1 (*n* = 1)	ST101 (1)	0	0	1	100	0	0	1	100	0	0	1	100

a OXA-48 group includes one OXA-48 + VIM-2-coproducing isolate.

b KPC-3 group includes one KPC-3 + VIM-2-coproducing isolate.

**FIG 4:**
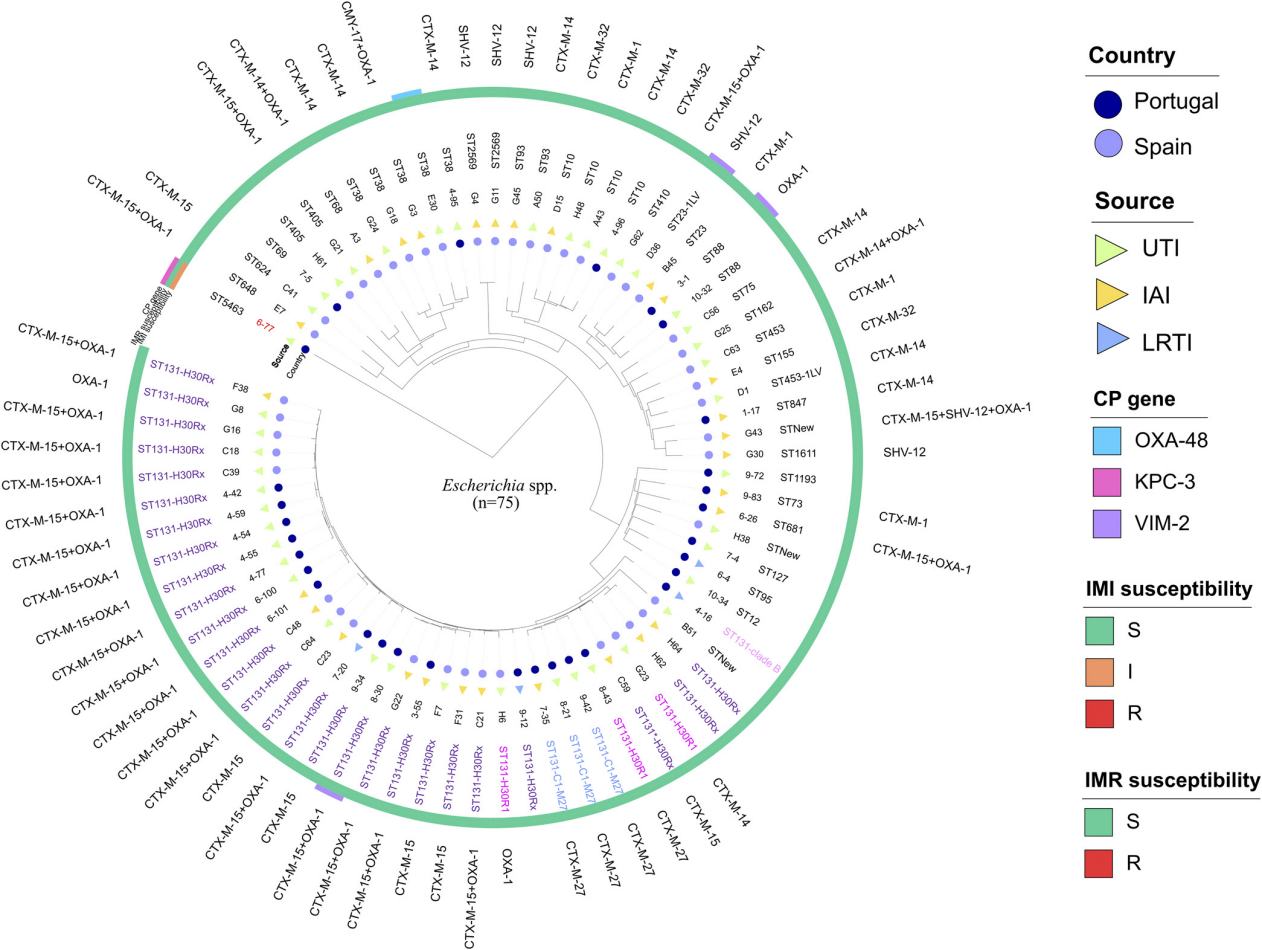
Similarity tree of Escherichia spp. isolates sequenced (*n* = 75) during the SUPERIOR and STEP surveillance studies and molecular data (sequence type, ST131 clade, and *bla* genes content) obtained by WGS. Imipenem-relebactam (IMR) (S, MIC ≤ 2/4 mg/liter; R, MIC > 2/4 mg/liter) and imipenem (IMI) (S, MIC ≤ 1 mg/liter; R, MIC > 4 mg/liter) susceptibility results interpreted according to EUCAST-2021 criteria are also included. Branch length is indicative of the MASH distance. E. coli species is shown in black letters and *E. marmotae* species is shown in red letters. E. coli isolates belonging to the ST131 high-risk clone are represented by colored letters: purple (ST131-H30Rx clade), dark pink (ST131-H30R1 clade), blue (ST131-C1-M27 subclade), and light pink (ST131 clade B).

In the sequenced Escherichia spp. collection (*n* = 75) (Spain [45 E. coli] and Portugal [29 E. coli and 1 Escherichia marmotae]), the presence of a carbapenemase gene was confirmed in 5 isolates (1 VIM-2-E. coli and 1 OXA-48-E. coli in Spain, 2 VIM-2-E. coli, and 1 KPC-3-*E. marmotae* in Portugal). All isolates were susceptible to both imipenem and imipenem-relebactam, although a reduction of 4-fold dilutions was observed in the imipenem-relebactam MIC value of the KPC-3-producing *E. marmotae* strain (MIC_IMI_ = 4 mg/liter [I] and MIC_IMR_ = 0.25/4 mg/liter [S]) (Fig. 4). ST131 was the most prevalent clone (45.3%, 34/75) in both Spain (37.8%, 17/45) and Portugal (56.7%, 17/30). The subclone ST131-B2-O25b:H4-H30Rx (clade 2) was predominant in both geographical regions (33.3% [15/45] in Spain and 40% [12/30] in Portugal) and was mostly associated with CTX-M-15 production (15/15 in Spain and 11/12 in Portugal). In addition, the sublineage CTX-M-27-producing ST131-H30 was also detected in Portuguese hospitals (13.3%, 4/30), and most of them (3/4) belonged to the C1-M-27 subclade. Among the non-CC131 E. coli isolates (54.7%, 41/75), a greater diversity of clones and ESBL enzymes was found in both countries (Fig. 4). Clinical, epidemiological, molecular, and antimicrobial susceptibility data are summarized in Table S3.

On the other hand, the imipenem-relebactam-resistant E. cloacae isolate (identified by WGS as Enterobacter hormaechei) (MIC_IMR_ > 64/4 mg/liter) belonged to the ST133 clone. Genes encoding VIM-1 and CTX-M-9 were confirmed in this isolate, together with a high content of resistance genes to other groups of antimicrobials, including the newly described mobile colistin resistance gene *mcr-9.1* (Table S4).

## DISCUSSION

Antimicrobial resistance is increasing worldwide and is of great concern in hospital settings. ICUs are the hospital wards with the highest prevalence of health care-associated infections (HAIs) and have higher rates of antimicrobial resistance than non-ICU wards due to the frequent use of antibiotics in critically ill patients, and different infection prevention and control practices (11, 12). According to data recovered in the ECDC annual epidemiological report for 2017, rates of resistance to carbapenems in Klebsiella spp. and E. coli isolates from HAI acquired in ICUs were 15.2% and 0.8%, respectively (10). In addition, both *Enterobacterales* species are often associated with a high level of coresistance to other antimicrobials groups, generating multidrug-resistant bacteria that cause severe infections with limited treatment options (13). For this reason, over the last decade, efforts have been focused on developing new drugs with activity against these multidrug-resistant Gram-negative pathogens.

In the SUPERIOR and STEP studies, susceptibility of imipenem-relebactam in the *Enterobacterales* collection was ≥ 97% in both Spanish and Portuguese ICUs. A previous survey performed in Spain in 2018 with *Enterobacterales* clinical isolates collected in 24 hospitals reported a lower imipenem-relebactam susceptibility rate (86%) (14). In our study, imipenem-relebactam (98.7%) and ceftazidime-avibactam (99.5%) susceptibility rates showed comparable values. In addition, as expected, ceftolozane-tazobactam also displayed good activity, except against the subset of carbapenemase-producing isolates. Note that recently described resistance to ceftazidime-avibactam mainly due to the emergence of novel KPC enzymes in K. pneumoniae high-risk clones after the antibiotic exposure questions the success of the clinical use of this novel combination for the treatment of severe infections caused by KPC carbapenemase producers (15, 16). In this sense, imipenem-relebactam has demonstrated to be a more clinically efficient alternative against *Enterobacterales* isolates carrying KPC-3, but also novel KPC variants, than ceftazidime-avibactam (14, 17).

In the SMART (Study for Monitoring Antimicrobial Resistance Trends) report, imipenem-relebactam showed MIC values ≤ 1 mg/liter (MIC interpreted using the imipenem CLSI breakpoint) in 94.8% of UTI, 95.9% of IAI and 94.5% of LRTI caused by *Enterobacterales* isolates from ICU wards (11). According to our results, comparable imipenem-relebactam susceptibility rates (MIC values ≤ 2 mg/liter, EUCAST criteria) were found in cUTI (99%), cIAI (98.1%), and LRTI (98.8%) from Spanish and Portuguese ICUs. Note that in our collection, imipenem-relebactam was especially active against Klebsiella spp. strains with carbapenemase phenotype recovered from LRTI (91.7%), only recovered from Portugal.

Overall, in the SUPERIOR and STEP studies, resistance rate to imipenem-relebactam in the *Enterobacterales* isolates was very low (1.3%) and was mostly due to the production of carbapenemases in Klebsiella spp. strains. In the Klebsiella spp. collection, imipenem-relebactam resistance rate was 3.6%, higher than in isolates collected in the United States as a part of the SMART study (0.3%) (18). Nevertheless, differences could be a consequence of the local epidemiology of carbapenemase-producing K. pneumoniae isolates in this country, where KPC producers are endemic (19, 20). An increased in the *in vitro* activity of imipenem with the addition of relebactam has been previously demonstrated against clinical KPC-*Enterobacterales* isolates in hospitals from Spain and UK (14, 21, 22). Coinciding with these studies, the addition of relebactam restored the imipenem susceptibility in all KPC-3-producing Klebsiella spp. from our collection. In fact, in the SUPERIOR and STEP studies, imipenem-relebactam resistance was mainly detected in OXA-48-producing Klebsiella spp., although the susceptibility level among OXA-48-K. pneumoniae remained high (67%). This high percentage could be a consequence of the low level of expression of resistance to imipenem that is often associated with OXA-48 type enzymes (23) but also to the different clinical breakpoint defined for imipenem (S, MIC_IMP_ ≤ 1 mg/liter) and imipenem-relebactam (S, MIC_IMR_ ≤ 2/4 mg/liter). Furthermore, most of OXA-181-K. pneumoniae producers were fully susceptible. Previous data have also demonstrated a moderated activity of imipenem-relebactam against isolates of both K. pneumoniae and E. coli with class D OXA-48 enzymes (21, 24). However, further studies should be performed to demonstrate if susceptible imipenem-relebactam MIC values obtained *in vitro* are predictive of clinical success against OXA-48-producing *Enterobacterales*.

The occurrence of OXA-48-producing Klebsiella spp. also explain the differences in the imipenem-relebactam resistance rate detected in both countries (2.2% in Spain and 0.5% in Portugal). In Spain, OXA-48 was the most frequent carbapenemase and was associated with K. pneumoniae belonging to the ST11 high-risk clone. The OXA-48-ST11-K. pneumoniae is the most widespread clone in Spanish hospitals and has been previously related to a moderate activity of imipenem-relebactam (14). In fact, resistance to imipenem-relebactam in Spain has been more frequently associated with the OXA-48-ST147-K. pneumoniae clone (14). In our study, the ST147-K. pneumoniae clone was mostly detected in Portuguese ICUs and was not linked to the OXA-48 production or imipenem-relebactam resistance. In Portugal, spread of KPC-3 enzyme was predominant and was detected in a wide variety of K. pneumoniae high-risk clones (ST13, ST15, ST11, ST405…) also previously related to nosocomial infections. Previous reports have registered a high dissemination of KPC enzymes among K. pneumoniae high-risk clones along Portuguese hospitals (25, 26). However, the STEP is the first surveillance performed in this country testing the activity of imipenem-relebactam and comparators against clinical *Enterobacterales* strains.

On the other hand and coinciding with a previous Spanish study (22), all E. coli isolates recovered from Spanish and Portuguese ICUs showed susceptible MIC values to imipenem and imipenem-relebactam, including those in which carbapenemase production (mostly VIM-2) was found. The high-risk clone ST131-H30Rx (clade 2) associated with CTX-M-15 production was the most prevalent E. coli strain in both countries. Additionally, three CTX-M-27-producing ST131-H30 that belonged to the C1-M-27 subclade were also found in Portugal. Coinciding with our results, Johnston et al. (24) demonstrated that imipenem-relebactam was highly active against clinical E. coli isolates recovered from across the United States, with a higher percentage of susceptibility among the ST131 high-risk clone, particularly in the H30R and H30Rx clades and in KPC-producing strains.

Multidrug resistance in *Enterobacterales* that cause severe infections in ICUs remains a serious challenge worldwide and requires different interventions, including stewardship programs, implementation of infection control measures, rapid diagnostic tools, and also the development of novel therapeutic options. Imipenem-relebactam is positioned as a treatment option against KPC-producing K. pneumoniae isolates frequently detected in ICU patients with complicated infections in which few or no other treatment options are available. Despite the elevated susceptibility rate detected in this study, OXA-48-producing K. pneumoniae high-risk clones widely disseminated in hospital settings in Spain are the main contributor to imipenem-relebactam resistance among multidrug-resistant *Enterobacterales* isolates causing complicated infections in ICU patients.

## MATERIALS AND METHODS

### Study design and selection of isolates.

STEP and SUPERIOR are two multicenter surveillance studies designed to assess the *in vitro* activity of ceftolozane-tazobactam, imipenem-relebactam, and comparators agents against *Enterobacterales* and P. aeruginosa clinical isolates prospectively collected from ICU patients with cUTI, cIAI or LRTI admitted to 8 Spanish (SUPERIOR, April 2016–April 2017) and 11 Portuguese (STEP, June 2017–July 2018) hospitals (27, 28). All *Enterobacterales* clinical isolates, except those belonging to the *Morganellaceae* family, were collected during the SUPERIOR (359 *Enterobacterales* [203 E. coli, 100 Klebsiella *spp.*, and 56 other *Enterobacterales*]) and STEP [(175 E. coli, 152 Klebsiella spp., and 61 other *Enterobacterales*)] studies were recovered to evaluate the activity of imipenem-relebactam and comparators (27, 28). The Ramón y Cajal University Hospital (Madrid, Spain) was the central laboratory for the microbiological study and the subsequent genome characterization. The SUPERIOR study was approved by the Spanish Medicines Agency (Ref. MSD-CEF-2016-01) and the Ethical Committee of the Hospital Universitario Ramón y Cajal (Ref. 087-16). The STEP study was approved by the Ethical Committees of all participating Portuguese Hospitals.

Up to 199 *Enterobacterales* isolates (SUPERIOR [45 E. coli and 44 Klebsiella spp. and 1 E. cloacae] and STEP [30 E. coli and 79 Klebsiella spp.]) were analyzed using the WGS approach. All *Enterobacterales* isolates that showed a reduced imipenem-relebactam *in vitro* activity (*n* = 10) following the EUCAST-2021 criteria (https://www.eucast.org/fileadmin/src/media/PDFs/EUCAST_files/Breakpoint_tables/v_11.0_Breakpoint_Tables.pdf) were included. Additionally, a representative subset of E. coli and Klebsiella spp. isolates, all of them susceptible to imipenem-relebactam, with ESBL (*n* = 135), carbapenemase (*n* = 38) and non-ESBL-noncarbapenemase (*n* = 16) phenotypes, previously characterized as a part of the SUPERIOR and STEP studies to research the ceftolozane-tazobactam resistance mechanisms, were also included in the genome analysis (Table S5) (29, 30).

### Antimicrobial susceptibility testing.

MIC values of imipenem-relebactam and comparators were determined at the central laboratory by the standard broth microdilution method (BMD) using frozen 96-well plates (Thermo Fisher Scientific, Cleveland, OH). The antimicrobial concentrations tested were as follows: ceftazidime-avibactam (CZA; 0.03/4-64/4 mg/liter), ceftolozane-tazobactam (CTZ; 0.03/4 to 32/4 mg/liter), imipenem-relebactam (IMR; 0.03/4-64/4 mg/liter), and imipenem (IMI; 0.03 to 64 mg/liter). E. coli ATCC 25922, E. coli ATCC 35218, and K. pneumoniae ATCC 700603 strains were used as quality control. Interpretation of results was performed following the European Committee on Antibiotic Susceptibility Testing (EUCAST-2021; https://www.eucast.org/fileadmin/src/media/PDFs/EUCAST_files/Breakpoint_tables/v_11.0_Breakpoint_Tables.pdf) and Clinical and Laboratory Standards Institute (CLSI-2021; https://clsi.org/standards/products/microbiology/documents/m100/) guidelines. The EUCAST-2021 breakpoints used for imipenem-relebactam were: susceptible (S, MIC_IMR_ ≤ 2/4 mg/liter) and resistant (R, MIC_IMR_ > 2/4 mg/liter). CLSI-2021 clinical breakpoints (CBPs) applied for imipenem-relebactam were as follows: S, MIC_IMR_ ≤ 1/4 mg/liter; and R, MIC_IMR_ ≥ 4/4 mg/liter.

### Whole-genome sequencing and bioinformatics analysis.

Genomic DNA extraction was performed using the commercial Chemagic DNA Bacterial External Lysis kit (PerkinElmer, USA). Short-read sequencing was performed using the Illumina Hiseq4000 or the Illumina NovaSeq 6000 platforms (OGC, Oxford, UK), with 2 × 150-bp paired-end reads. Sequencing processing, molecular typing, and antibiotic resistance prediction were carried out as previously described (29, 30).

### Sequence data.

All complete sequences were deposited at DDBJ/ENA/GenBank under the following BioProject accession numbers: PRJNA609897 (SUPERIOR study) and PRJNA602991 (STEP study) (Table S6).
