# Supplementary material for: Imipenem-Relebactam Susceptibility in Enterobacterales Isolates Recovered from ICU Patients from Spain and Portugal (SUPERIOR and STEP Studies)
Source: Microbiol Spectr. 2022 Aug 31;10(5):e02927-22. doi: 10.1128/spectrum.02927-22 (PMC9602286; doi:10.1128/spectrum.02927-22)
Supplement: Supplemental file 1 — Tables S1 and S5 and Fig. S1 and S2. Download spectrum.02927-22-s0001.pdf, PDF file, 0.3 MB [file spectrum.02927-22-s0001.pdf]

**Table S1.** Distribution of *Enterobacterales* (except *Morganellaceae*) clinical isolates recovered during the SUPERIOR and STEP surveillance studies by species and infection type.

| Genera | Species | Spain (SUPERIOR) | | | Portugal (STEP) | | | | TOTAL |
|---|---|---|---|---|---|---|---|---|---|
| | | **cUTI** | **cIAI** | **Total** | **cUTI** | **cIAI** | **LRTI** | **Total** | |
| ***Escherichia* spp.** | ***Escherichia coli*** | **125** | **78** | **203** | **108** | **51** | **16** | **175** | **378** |
| ***Klebsiella* spp.** | **Total** | **55** | **45** | **100** | **67** | **38** | **47** | **152** | **252** |
| | *Klebsiella pneumoniae* | 48 | 38 | 86 | 60 | 34 | 31 | 125 | 211 |
| | *Klebsiella aerogenes* | 2 | 3 | 5 | 6 | 3 | 11 | 20 | 25 |
| | *Klebsiella oxytoca* | 4 | 4 | 8 | 1 | 1 | 3 | 5 | 13 |
| | *K. variicola* | 0 | 0 | 0 | 0 | 0 | 2 | 2 | 2 |
| | *Raoultella ornithinolytica* | 1 | 0 | 1 | 0 | 0 | 0 | 0 | 1 |
| ***Enterobacter* spp.** | **Total** | **15** | **13** | **28** | **9** | **15** | **12** | **36** | **64** |
| | *Enterobacter cloacae* | 15 | 11 | 26 | 7 | 15 | 12 | 34 | 60 |
| | *Enterobacter kobei* | 0 | 1 | 1 | 0 | 0 | 0 | 0 | 1 |
| | *Enterobacter asburiae* | 0 | 1 | 1 | 1 | 0 | 0 | 1 | 2 |
| | *Enterobacter hormaechei* | 0 | 0 | 0 | 1 | 0 | 0 | 1 | 1 |
| ***Serratia* spp.** | **Total** | **5** | **4** | **9** | **4** | **4** | **7** | **15** | **24** |
| | *Serratia marcescens* | 5 | 4 | 9 | 4 | 3 | 7 | 14 | 23 |
| | *Serratia liquefasciens* | 0 | 0 | 0 | 0 | 1 | 0 | 1 | 1 |
| ***Citrobacter* spp.** | **Total** | **3** | **6** | **9** | **3** | **3** | **4** | **10** | **19** |
| | *Citrobacter koseri* | 1 | 2 | 3 | 3 | 1 | 3 | 7 | 10 |
| | *Citrobacter freundii* | 1 | 2 | 3 | 0 | 1 | 0 | 1 | 4 |
| | *Citrobacter braaki* | 1 | 1 | 2 | 0 | 1 | 1 | 2 | 4 |
| | *Citrobacter sakazakii* | 0 | 1 | 1 | 0 | 0 | 0 | 0 | 1 |
| ***Hafnia* spp.** | ***Hafnia alvei*** | 1 | 4 | **5** | 0 | 0 | 0 | **0** | **5** |
| ***Providencia spp.*** | ***Providencia stuartii*** | 3 | 0 | **3** | 0 | 0 | 0 | **0** | **3** |
| ***Kluyvera* spp.** | ***Kluyvera ascorbata*** | 1 | 0 | **1** | 0 | 0 | 0 | **0** | **1** |
| ***Salmonella* spp.** | ***Salmonella enterica*** | 0 | 1 | **1** | 0 | 0 | 0 | **0** | **1** |
| TOTAL *Enterobacterales* | | **208** | **151** | **359** | **191** | **111** | **86** | **388** | **747** |

cUTI= complicated urinary tract infection; cIAI= complicated intra-abdominal tract infection; LRTI= lower respiratory tract infection

**Table S5.** *Enterobacterales* isolates selected for whole genome sequencing.

| | | IMR MIC (mg/L) | | | IMR[1] phenotype | | |
|---|---|---|---|---|---|---|---|
| **Bacterial spp.** | **Phenotype** | **Range** | **$MIC_{50}$** | **$MIC_{90}$** | **Susceptible** | **Resistant** | **TOTAL** |
| *Klebsiella spp.* | ESBL[2] | 0.06/4-0.5/4 | 0.12/4 | 0.5/4 | 62 | 0 | 62 |
| | CP[3] | 0.06/6->64/4 | 1/4 | 64/4 | 36 | 9 | 45 |
| | non-ESBL non-CP | 0.12/4-0.25/4 | 0.25/4 | 0.25/4 | 16 | 0 | 16 |
| | TOTAL | 0.06/4->64/4 | 0.25/4 | 2/4 | 114 | 9 | 123 |
| *E. coli* | ESBL | 0.12/4-2/4 | 0.12/4 | 0.25/4 | 73 | 0 | 73 |
| | CP | 0.12/4-0.25/4 | - | - | 2 | 0 | 2 |
| | TOTAL | 0.12/4-2/4 | 0.12/4 | 0.25/4 | 75 | 0 | 75 |
| *Enterobacter cloacae* | CP | >64/4 | - | - | 0 | 1 | 1 |
| TOTAL | | | | | **189** | **10** | **199** |

ESBL= extended spectrum-β-lactamase; CP= carbapenemase
[1] IMR resistance phenotype (MICs > 2/4 mg/L, EUCAST-2021 criteria).
[2] ESBL phenotype (MICs ≥2 mg/L for cefotaxime, ceftazidime and/or cefepime).
[3] CP phenotype (MICs > 1 mg/L for imipenem and/or > 0.12 mg/L for meropenem).

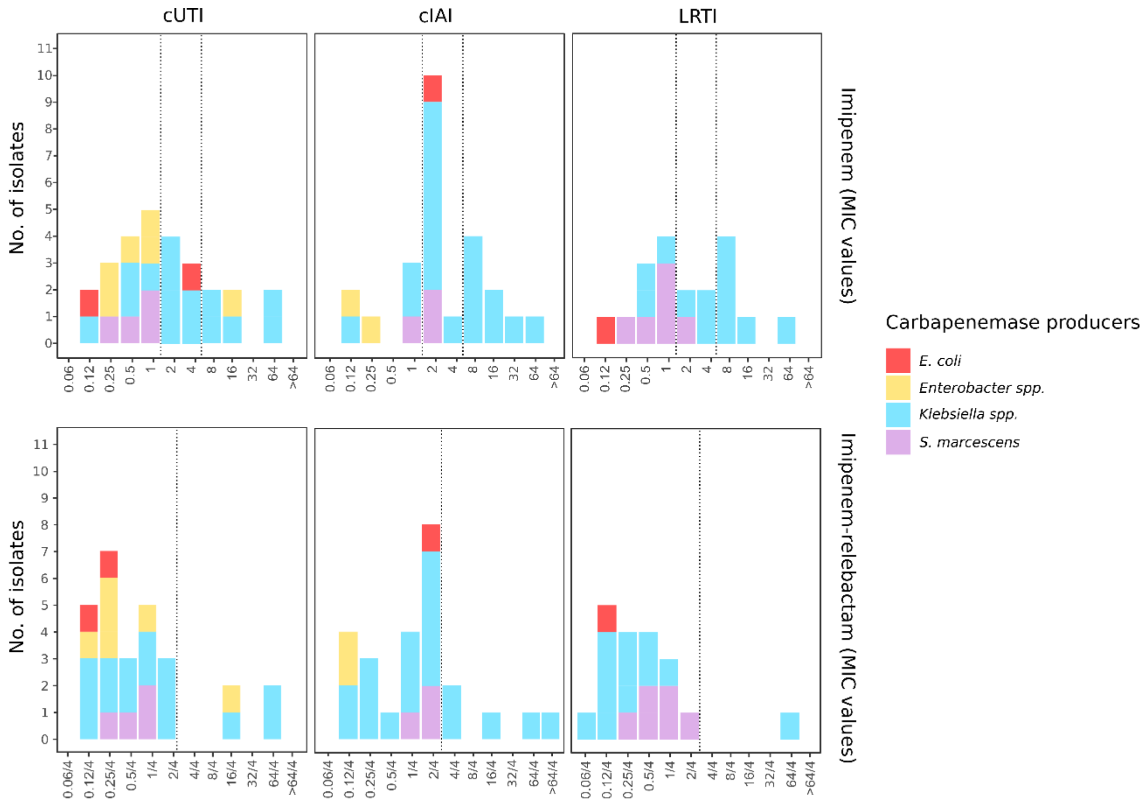


**Figure S1.** Distribution of carbapenemase-producing *Enterobacterales* isolates (n=69) recovered during the SUPERIOR and STEP surveillance studies by the MIC value of imipenem-relebactam and imipenem and by infection source (cUTI= complicated urinary tract infection; cIAI= complicated intra-abdominal tract infection; LRTI= lower respiratory tract infection). Dotted lines represent the EUCAST clinical breakpoints of imipenem-relebactam (S, MIC ≤ 2/4 mg/L; R, MIC > 2/4 mg/L) and imipenem (S, MIC ≤ 1 mg/L; R, MIC > 4 mg/L).

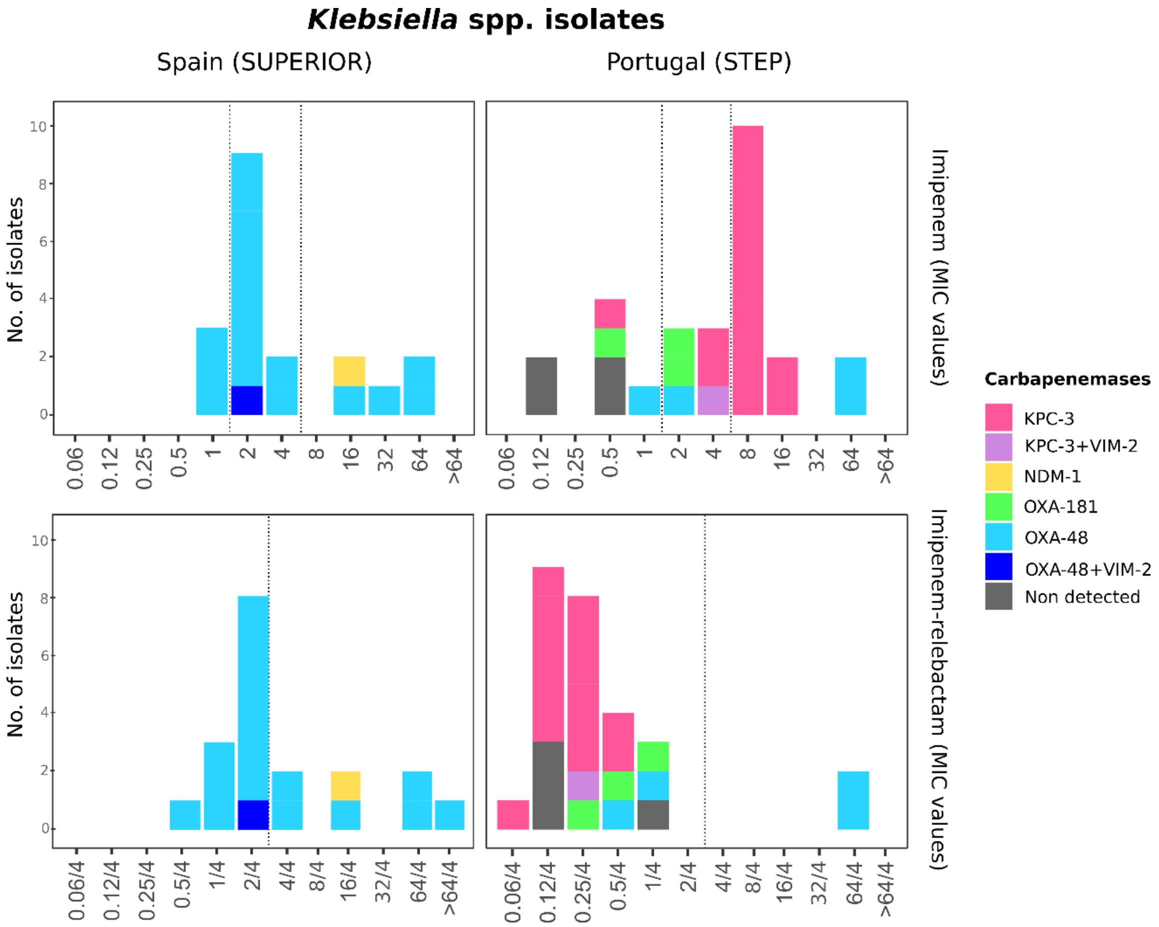


**Figure S2.** Distribution of sequenced *Klebsiella* spp. isolates with a carbapenemase phenotype (n=46) recovered during the SUPERIOR and STEP surveillance studies by the MIC value of imipenem-relebactam and imipenem and the carbapenemase type detected. Dotted lines represent the EUCAST clinical breakpoints of imipenem-relebactam (S, MIC ≤ 2/4 mg/L; R, MIC > 2/4 mg/L) and imipenem (S, MIC ≤ 1 mg/L; R, MIC > 4 mg/L).
